# NAC047/052/104 Synergistically Regulate the Dark-Induced Leaf Senescence in Non-Heading Chinese Cabbage

**DOI:** 10.3390/ijms26052340

**Published:** 2025-03-06

**Authors:** Bing Yang, Dingyu Zhang, Zitong Meng, Yijiang Yin, Xiao Yang, Mengqin Cao, Ruixin Li, Yishan Song, Hongfang Zhu

**Affiliations:** 1 College of Food Science and Technology, Shanghai Ocean University, Shanghai 201306, China; y17739190411@163.com (B.Y.); 17797622297@163.com (Z.M.); 18536495112@163.com (M.C.); 2 Shanghai Key Laboratory of Protected Horticultural Technology, Horticultural Research Institute, Shanghai Academy of Agricultural Sciences, Shanghai 201403, China; zhangdy1225@126.com (D.Z.); 13577597931@163.com (Y.Y.); yangxiao2113@163.com (X.Y.); l13368613997@outlook.com (R.L.)

**Keywords:** NHCC, dark, NACs, leaf senescence

## Abstract

Non-heading Chinese cabbage (NHCC) is an important vegetable, and its leaves are harvested for consumption. Thus, the initiation and progression of leaf senescence in NHCC directly impact its yield and quality. In multiple plant species, NAC transcription factors are known to act as critical regulators of leaf senescence. However, in NHCC, the NAC transcription factors contributing to leaf senescence regulation remain to be identified, and the mechanisms underlying dark-induced leaf senescence remain unclear. To explore the molecular mechanisms of leaf senescence in NHCC, we stored NHCC away from light and subsequently examined dark-induced transcriptional alterations via RNA sequencing. Interestingly, three *NAC* transcription factors, *BrNAC047*, *BrNAC052*, and *BrNAC104*, were found to be potently activated by darkness. Subsequently, the virus-induced gene silencing of *BrNAC047*, *BrNAC052*, and *BrNAC104* demonstrated that these three *NACs* act as positive regulators of dark-induced leaf senescence in NHCC. Dual-luciferase assays further confirmed that BrNAC047, BrNAC052, and BrNAC104 directly activate the promoters of certain senescence-associated genes. This study uncovers the molecular signaling pathways governing dark-induced leaf senescence in NHCC, highlighting the role of three key regulators and offering valuable molecular targets for delaying leaf senescence in NHCC.

## 1. Introduction

Non-heading Chinese cabbage (NHCC; *Brassica rapa*) is extensively cultivated throughout Asia [1,2]. The leaves of this plant act as distinctive autotrophic apparatuses, converting light energy into energy substrates such as sugar via photosynthesis [3,4]. However, when the leaves enter the senescence phase, the disintegration of chloroplasts triggers the efflux of nutrients into the nascent parts of the plant, causing the leaves to wither and undergo de-greening [5,6]. Moreover, leaf senescence is not only a developmental phenomenon but also a direct response to external stress in these plants [7].

Leaf senescence is regulated by a series of senescence-associated genes (SAGs). The transcriptional activity of SAGs increases leaf age and is strictly controlled by multi-level regulatory mechanisms [8,9,10]. Leaf senescence is thus a consequence of the combined regulatory effects of both endogenous and exogenous environmental elements [11,12]. Notably, light is the primary external factor modulating leaf senescence. Light is not only essential for energy production via photosynthesis, facilitating plant growth, but also operates as a signal to regulate the development and life cycle of plants [13]. During post-harvest storage, NHCC is inevitably exposed to dark conditions for prolonged periods, which induces rapid leaf decay. Studies have demonstrated that dark conditions trigger rapid senescence in plants, and dark treatment is thus a common approach for studying leaf senescence across multiple plants [14,15]. Hence, investigations into the molecular machinery regulating dark-induced senescence in NHCC could be valuable for resolving the quality deterioration issues associated with storage.

Transcription factors regulate plant growth, development, and stress responses by modulating downstream functional genes [16]. The NAC family is among the largest and most important families of transcription factors in plants [17]. NAC proteins contain a highly conserved NAC domain, which functions as a transcriptional regulator [18]. In model plants, such as *Arabidopsis thaliana* and rice, the expression of NAC transcription factors is correlated with stress responses and senescence regulation [19,20,21]. Specifically, in *A. thaliana*, the germination capacity of *AtNAC092* knockout and overexpression lines appears to be augmented and diminished under salt stress, respectively [22]. Meanwhile, the upregulation of *ATNAP* can accelerate leaf senescence in this species [23]. Similarly, *TaNAC29* expression enhances the antioxidant potency of wheat plants, diminishes the accumulation of hydrogen peroxide (H_2_O_2_), and mitigates membrane impairment, thereby improving the resistance of wheat to salt and drought stresses [24,25]. Additionally, NAC transcription factors also regulate phytohormone signals to regulate leaf senescence. *ANAC019*, *ANAC055*, and *ANAC072* positively regulate abscisic acid (ABA)-induced leaf senescence in *A. thaliana*. In addition, ATNAP interacts with SAG113, further mediating and regulating ABA-triggered leaf senescence [26,27,28]. These studies highlight the pivotal functions of NAC transcription factors in leaf senescence and stress responses. Nevertheless, which NAC transcription factors modulate the progression of leaf senescence in NHCC remains unclear, and their potential mechanisms of action are yet to be delineated.

In this study, we investigated the mechanism of leaf senescence in NHCC by incubating detached leaves under dark conditions. Through transcriptomic analysis, we identified some NAC transcription factors that act as cardinal responsive genes during dark induction in NHCC. Subsequently, virus-induced gene silencing (VIGS) assays revealed that *BrNAC047*, *BrNAC052*, and *BrNAC104* play a positive role in regulating dark-induced leaf senescence in NHCC. Molecular assays confirmed that BrNAC047, BrNAC052, and BrNAC104 can directly activate the promoters of several SAGs, thereby accelerating leaf senescence in NHCC. The present study not only uncovers some novel NAC transcription factors involved in modulating leaf senescence but also delineates the molecular mechanisms underlying dark-induced leaf senescence in NHCC.

## 2. Results

### 2.1. Phenotypic and Physiological Analysis of Dark-Induced Senescence in NHCC

**Figure 1:**
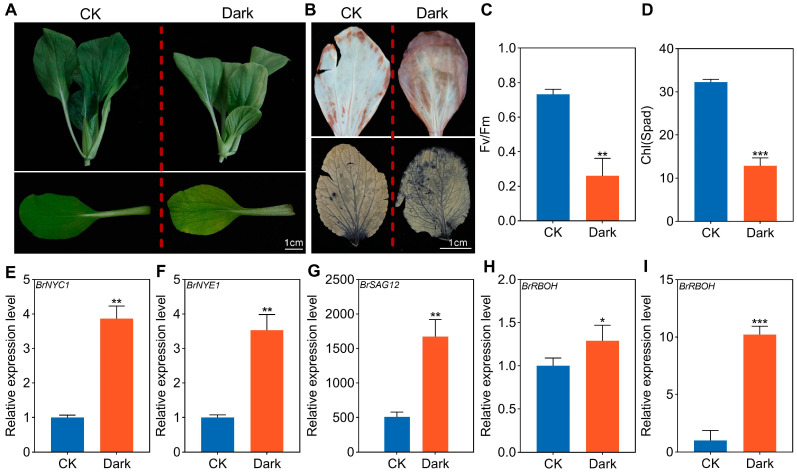
Phenotypes of dark-treated NHCC leaves. (**A**) Phenotypes of NHCC leaves after dark treatment; (**B**) DAB and NBT staining of NHCC leaves after dark treatment; (**C**) F_V_/F_m_ of NHCC leaves after dark treatment; (**D**) Chlorophyll content of NHCC leaves after dark treatment; (**E**–**I**) Expression levels of *BrNYC1*, *BrNYE1*, *BrSAG12* and *BrRBOHs* in NHCC leaves after dark treatment. Processing time: 3 days. Data represent the mean ± SD (*n* = 3 biological replicates), *** *p* < 0.001, ** *p* < 0.01, and * *p* < 0.05 (*t*-test).

To uncover the regulatory mechanism underlying dark-induced leaf senescence in NHCC, we incubated NHCC leaves in the dark. As shown in Figure 1, significant differences in leaf color emerged in NHCC after 3 days of dark treatment. Specifically, the leaves started to turn yellow due to chlorophyll degradation (Figure 1A). DAB and NBT staining assays indicated that, compared with the control group, the treatment group exposed to darkness contained higher accumulated levels of ROS (Figure 1B). Moreover, both the photosynthetic function and chlorophyll content of the leaves displayed a marked downward trend following dark treatment (Figure 1C,D). Expression analyses of the key enzymes involved in the chlorophyll degradation and ROS biosynthesis pathways revealed that the chlorophyll catabolism genes *NYC1* (*BrC04g009380*), *SAG12 (BrC02g034440)* and *NYE1* (*BrC03g052200*), along with the ROS-producing enzyme genes *RBOH* (*BrC02g041610*) and *RBOH* (*BrC03g016000*), were significantly upregulated in the dark treatment group (Figure 1E–I). Collectively, these results demonstrated that dark treatment promotes the degradation of chlorophyll in NHCC leaves, accelerates the biosynthesis of ROS, and thus accelerates the leaf senescence process.

### 2.2. Analysis of Dark-Induced Transcriptomic Alterations in NHCC Leaves

**Figure 2:**
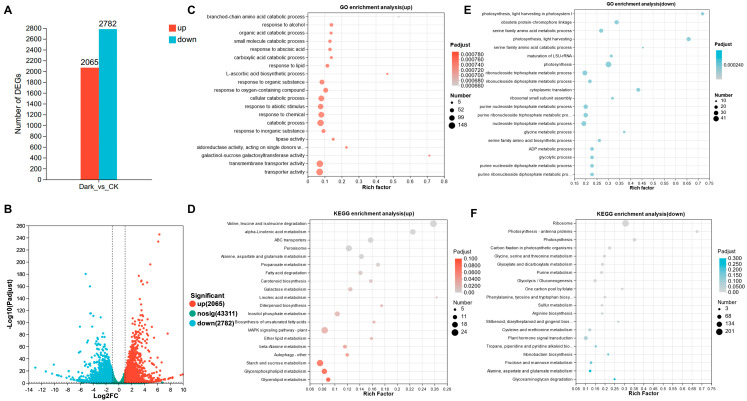
Overview of transcriptome data and analysis of signaling pathways triggered by dark treatment in NHCC: (**A**) Statistical analysis of differentially expressed genes (DEGs) in NHCC leaves after dark treatment; (**B**) volcano plot showing the DEGs in NHCC leaves after dark treatment; (**C**,**D**) GO and KEGG analyses of the upregulated DEGs in NHCC leaves; and (**E**,**F**) GO and KEGG analyses of the downregulated DEGs in NHCC leaves. Data represent the mean ± SD (n = 3 biological replicates). Full name of the pathway in the diagram: oxidoreductase activity, acting on single donors with incorporation of molecular oxygen, incorporation of two atoms of oxygen; photosynthesis, light harvesting in photosystem II; purine ribonucleotide triphosphate metabolic process; purine ribonucleotide metabolic process; phenylanine, tyrosine and gingerol biosynthesis; Stilbenoid, diarylheptanoid, and gingerol biosynthesis; Tropane, Piperidine, and Pyridine Alkaloid Biosynthesis.

To explore the molecular signaling pathways involved in dark-induced leaf senescence, we performed transcriptomic sequencing analysis on NHCC leaves following dark treatment. The data revealed that dark treatment triggered drastic transcriptional changes in NHCC leaves. Specifically, after dark treatment, 2605 genes were significantly upregulated, while 2782 genes were significantly downregulated (Figure 2A,B). Using Gene Ontology (GO) analysis, we found that the upregulated genes were associated with the abscisic acid pathway, organic acid catabolism pathway, and response to abiotic stimuli in NHCC. Meanwhile, the downregulated genes were associated with pathways such as photosynthesis and glycolysis (Figure 2C,E). Through the Kyoto Encyclopedia of Genes and Genomes (KEGG) analysis of the DEGs, we found that the upregulated genes were mainly enriched in signaling pathways such as amino acid metabolism, fatty acid degradation, and carotenoid biosynthesis. In contrast, the downregulated genes were primarily linked to light signaling, including photosynthesis antenna proteins and photosynthesis (Figure 2D,F). Collectively, these findings offer compelling proof of the significant molecular differences between NHCC leaves receiving light and those stored under dark conditions.

### 2.3. Darkness Primarily Activates NAC Transcription Factors in NHCC

**Figure 3:**
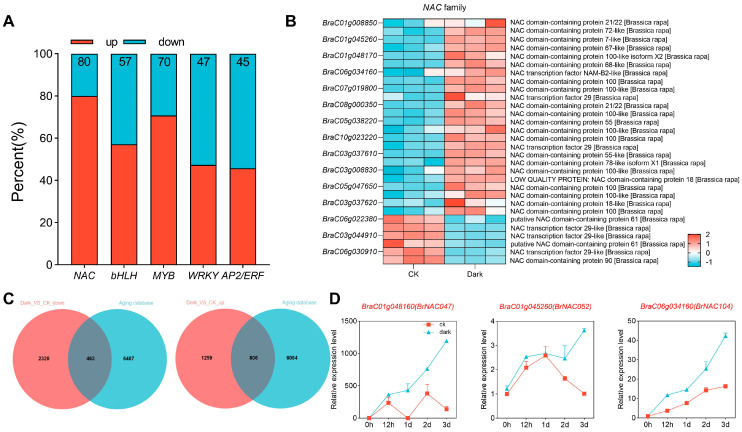
Dark-induced signal transduction involves *NAC* transcription factors. (**A**) A 100% stacked bar chart, showing the dark-induced transcription factors in NHCC leaves. (**B**) Heatmap of dark-induced *NAC* family genes in NHCC leaves. (**C**) Venn analysis comparing differentially expressed genes with those from the leaf senescence gene database. (**D**) Expression profiles of *BrNAC047*, *BrNAC052*, and *BrNAC104* in the control and dark treatment groups. DEGs were defined based on the following thresholds: Log2 (foldchange) ≥ 1, FDR < 0.05 (*n* = 2 biological replicates). Data represent the mean ± SD (*n* = 3 biological replicates).

Given that dark treatment induced considerable transcriptomic variations in NHCC, we further analyzed the response of various transcription factor families to darkness in this species. The *NAC*, *bHLH*, *MYB*, *WRKY*, and *AP2/ERF* families are the most important transcription factor families in plants. By examining the expression patterns of these transcription factors, we observed that the vast majority of *NAC* transcription factors were significantly upregulated in NHCC leaves after dark treatment. Similarly, most *MYB* transcription factors also showed a trend of upregulation following dark treatment. In contrast, the distribution of upregulated and downregulated *bHLH*, *AP2/ERF*, and *WRKY* transcription factors was comparable. Collectively, these findings suggested that *NAC* transcription factors are likely to play a decisive role in dark-induced leaf senescence (Figure 3A,B).

Subsequently, a Venn diagram analysis was conducted by comparing the DEGs altered by dark treatment with those in the leaf senescence gene database. Notably, of the dark-induced SAGs, 806 genes exhibited an upregulated expression profile and 463 exhibited a downregulated expression profile (Figure 3C). Notably, 13 *NAC* genes were prominently activated and showed significant transcriptional elevation following dark treatment (Figure S1). The expression analysis of these genes revealed that most of them exhibited elevated expression levels 3 days after dark treatment. Three genes in particular—*BraC01g048160* (*BrNAC047*), *BraC01g045260* (*BrNAC052*), and *BraC06g034160* (*BrNAC104*)—were exceptionally sensitive to darkness and remained in an activated state under dark conditions (Figure 3D). These findings strongly implied that these three *NAC* genes may play a cardinal role in dark-induced leaf senescence in NHCC.

### 2.4. Molecular Characterization of BrNAC047, BrNAC052, and BrNAC104

**Figure 4:**
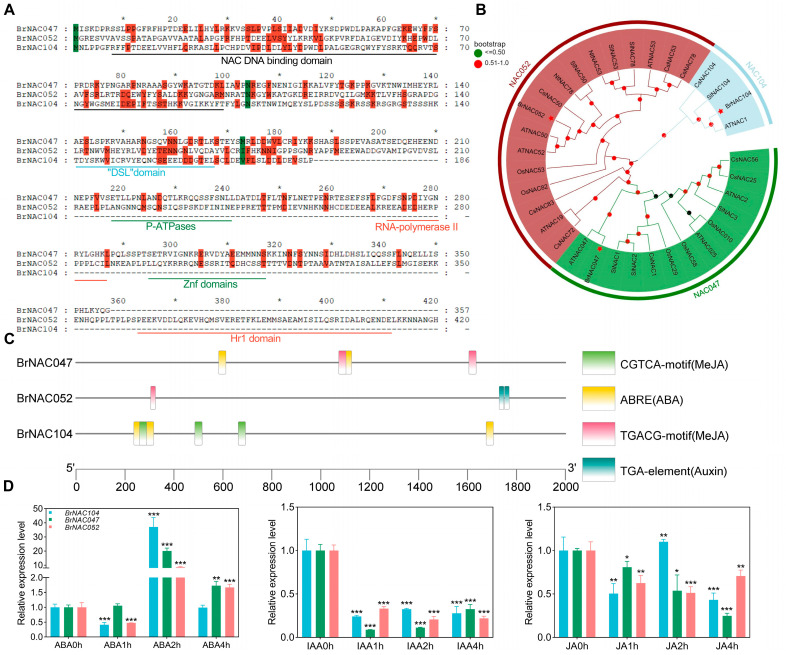
Protein and DNA sequence analyses of BrNAC047, BrNAC052, and BrNAC104. (**A**) Protein structure prediction of BrNAC047, BrNAC052, and BrNAC104. * Number is an omission of the number of amino acid sequences. The green color indicates the same amino acids, while the red color represents unique amino acids. (**B**) Phylogenetic analysis of BrNAC047, BrNAC052, and BrNAC104 in different species. Specially marked with a red asterisk. Multiple alignment was performed CLUSTALW, and the phylogenetic tree was constructed with MEGA6.0 using a bootstrap test of phylogeny with the neighbor-joining test and default parameters. (**C**) Analysis of the promoter sequences of *BrNAC047*, *BrNAC052*, and *BrNAC104*. (**D**) qPCR analysis of the responses of *BrNAC047*, *BrNAC052*, and *BrNAC104* to different phytohormones. Data represent the mean ± SD (*n* = 3 biological replicates). *** *p* < 0.001, ** *p* < 0.01, and * *p* < 0.05 (*t*-test).

BrNAC047, BrNAC052, and BrNAC104 appeared to be positive regulators of leaf senescence in NHCC. Hence, we conducted an in-depth comparative analysis of their molecular characteristics and upstream regulators. Amino acid sequence analysis shows that all these proteins contain highly conserved NAC DNA binding domains and other specific protein domains (Figure 4A). Subsequently, phylogenetic trees also confirmed that BrNAC047, BrNAC052, and BrNAC104 were closely related to ATNAC47, ATNAC052, and ATNAC1 (Figure 4B), respectively. Using the plantCARE and TBtools 2.136 software, a predictive analysis of the 1000 bp promoter sequences upstream of these genes was performed. The promoters of these three genes were found to contain several cis-regulatory elements that are potentially involved in responses to MeJA, ABA, and auxin (Figure 4C). Thus, we examined the induction of these three genes in response to different phytohormones via qPCR. Upon the exogenous application of ABA for 2 h, all three of these *NACs* showed pronounced upregulation (Figure 4D). This implied that ABA may play a crucial role in the dark-induced expression of *BrNAC047*, *BrNAC052*, and *BrNAC104* in NHCC.

### 2.5. BraNAC047/BrNAC052/BrNAC104 Positively Regulate Dark-Induced Leaf Senescence in NHCC

**Figure 5:**
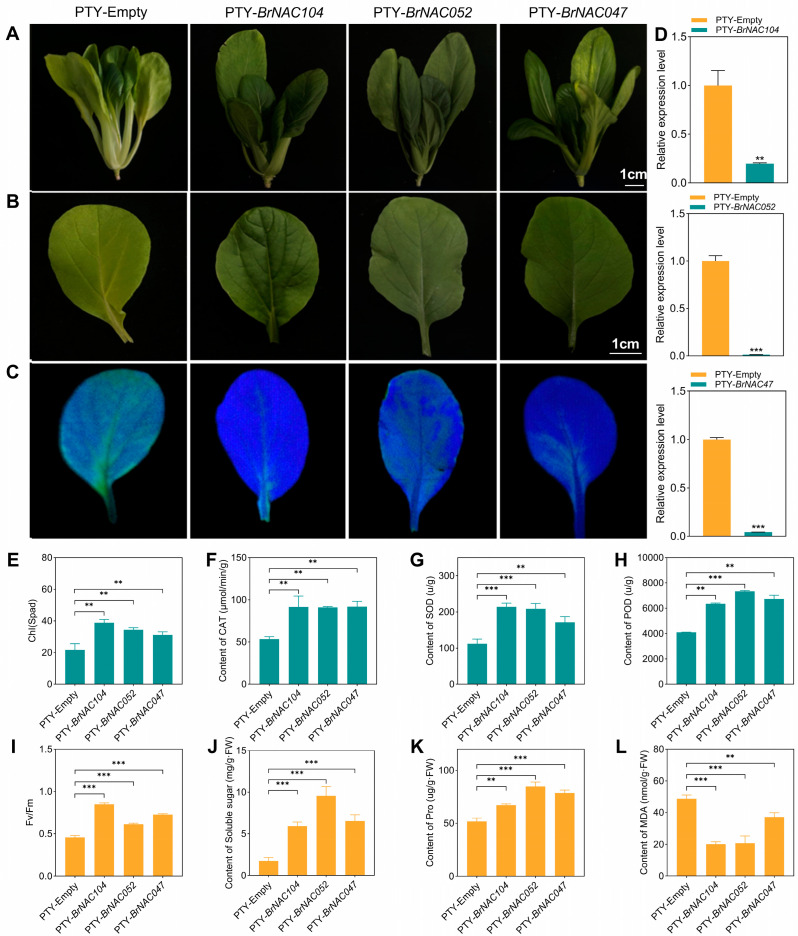
Functional validation of BrNAC047, BrNAC052, and BrNAC104 during dark-induced leaf senescence in NHCC. (**A**) Phenotypes of gene-silenced NHCC after dark treatment. (**B**) Phenotype of the first true leave from gene-silenced NHCC after dark treatment. (**C**) Chlorophyll fluorescence in the first true leave from the gene-silenced NHCC after dark treatment. (**D**) Gene expression levels of *NACs* in the gene-silenced NHCC plants. (**E**) Chlorophyll content in the gene-silenced NHCC leaves after dark treatment. (**F**–**H**) CAT, SOD, and POD contents in the gene-silenced NHCC leaves after dark treatment. (**I**) Photosynthetic capacity of the gene-silenced NHCC leaves after dark treatment. (**J**–**L**) Soluble sugar, proline, and MDA contents in the gene-silenced NHCC leaves after dark treatment. Data represent the mean ± SD (*n* = 3 biological replicates). *** *p* < 0.001, ** *p* < 0.01 (*t*-test).

To investigate whether *BrNAC047*, *BrNAC052*, and *BrNAC104* participate in the regulation of dark-induced leaf senescence in NHCC, we conducted VIGS experiments to inhibit *BrNAC047*, *BrNAC052*, and *BrNAC104* expression in NHCC. qPCR assays confirmed the knockdown of these *NAC* genes after VIGS (Figure 5D). After incubation in the dark for 3 days, the *BrNAC047-*, *BrNAC052-*, and *BrNAC104*-silenced plants and leaves exhibited delayed senescence when compared with control samples (Figure 5A–C). The gene-silenced leaves also displayed relatively higher chlorophyll levels and more robust photosynthesis capacities (Figure 5E,I). Enzyme assays demonstrated that following dark treatment, the activities of POD, CAT, and SOD in *BrNAC047-*, *BrNAC052-*, and *BrNAC104*-silenced leaves were significantly higher than those in the control leaves (Figure 5F–H). In addition, the proline and soluble sugar contents were significantly higher in the *BrNAC047-*, *BrNAC052-* and *BrNAC104*-silenced plants, while the MDA contents were lower (Figure 5J–L). Collectively, these findings demonstrated that BrNAC047, BrNAC052, and BrNAC104 positively regulate the senescence of NHCC by targeting multiple metabolic pathways.

### 2.6. BrNAC047, BrNAC052, and BrNAC104 Activate SAG Expression in NHCC

**Figure 6:**
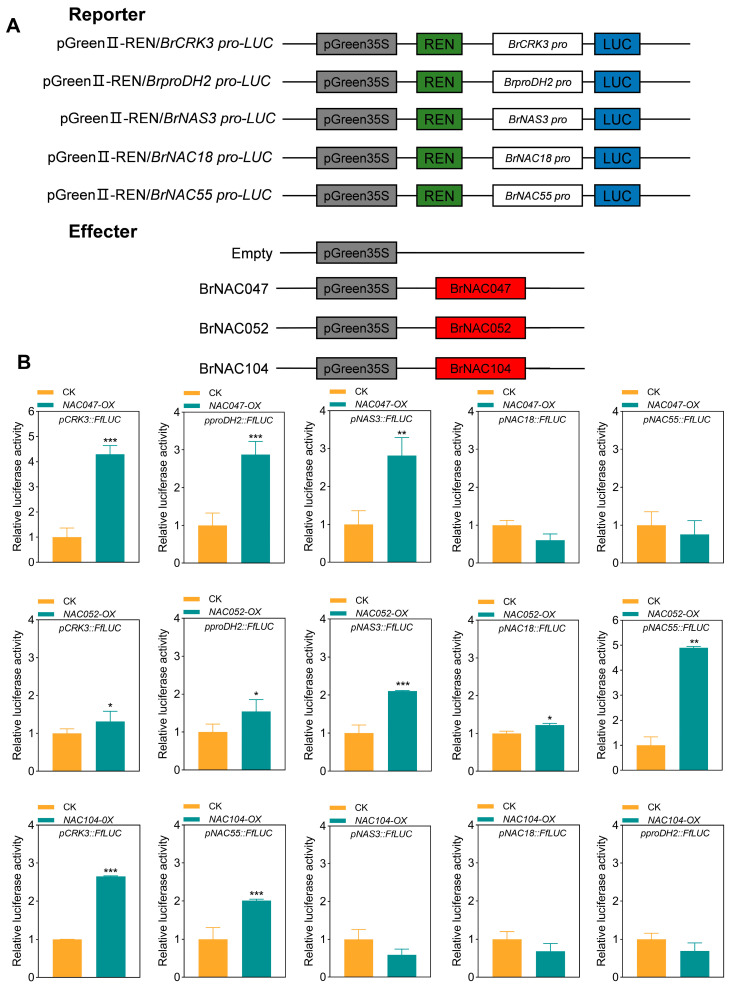
Dual-luciferase assay showing the effects of BrNAC047, BrNAC052, and BrNAC104 on the promoters of SAGs. (**A**) Schematics of the reporter and effector vectors. (**B**) Dual-luciferase transient expression assay in NHCC leaves showing that *BrNAC047*, *BrNAC052*, and *BrNAC104* activate the transcription of *pCRK3*, *pproDH2*, *pNAS3*, *pNAC18*, and *pNAC55*. The reporter and effector vectors are illustrated in the top panel. Data represent the mean ± SD (*n* = 3 biological replicates). *** *p* < 0.001, ** *p* < 0.01, and * *p* < 0.05 (*t*-test).

To explore whether BrNAC047, BrNAC052, and BrNAC104 directly regulate the expression of SAGs, we performed dual-luciferase assays in a tobacco model system to assess the regulatory effects of these NACs on the promoters of certain SAGs, namely, *CRK3*, *proDH2*, *NAS3*, *NAC18*, and *NAC55*. The promoters of *CRK3*, *proDH2*, *NAS3*, *NAC18*, and *NAC55* were cloned into pGreenII 0800-LUC vectors. Subsequently, these constructs were co-transfected along with *BrNAC047*, *BrNAC052*, and *BrNAC104* overexpression vectors into tobacco leaf tissues via *Agrobacterium tumefaciens*-mediated gene transfer (Figure 6A). As shown in Figure 6B, the overexpression of *BrNAC047*, *BrNAC052*, and *BrNAC104* significantly activated the promoters of the target SAGs. These confirmed that BrNAC047, BrNAC052, and BrNAC104 act as positive regulators of leaf senescence by directly controlling the expression of various SAGs.

## 3. Discussion

Darkness is an important factor inducing the deterioration of green leafy vegetables during the post-harvest stage. In model plants, such as *A. thaliana* and rice, the mechanism of dark-induced leaf senescence has been studied extensively. Nevertheless, how dark environments induce leaf senescence in NHCC has so far remained unclear [29,30,31]. In the present study, NHCC was exposed to the dark for a prolonged period. The results showed that dark treatment accelerates the de-greening of NHCC leaves. In addition, the expression levels of chlorophyll catabolism genes also exhibited remarkable upregulation (Figure 1A–F). Furthermore, ROS levels in NHCC leaves were substantially elevated after dark treatment (Figure 1B), and the genes encoding the ROS-generating enzyme *RBOH* were activated during the senescence process (Figure 1G–I). These findings suggested that darkness was indeed a key initiator of rapid leaf decay and quality loss in NHCC at the post-harvest stage.

Several studies have demonstrated that light is a pivotal environmental determinant governing the progression of leaf senescence [32]. Phytochrome-interacting factors (PIFs), which are key regulatory molecules, orchestrate the cellular responsiveness to light and temperature oscillations in plants [33]. Furthermore, phytochrome B (phyB) plays a critical role in plant growth and development, exerting a significant regulatory influence on the function of PIF4/5 [34,35]. Under conditions of appropriate illumination, phyB remains in an activated state and retains its capacity to repress the transcriptional function of PIF4/5, thereby sustaining the regular growth cadence of plants and maintaining morphogenetic processes. This regulatory mechanism has a very important position within the intricate network of plant photomorphogenesis and environmental acclimatization [36]. However, we did not observe any conspicuous upward trend in *PIF* expression under dark conditions in the present study, and the expression of *PIFs* appeared to be unresponsive to dark treatment (Figure S2). These results implied that the responsiveness of PIFs to light mainly occurs at the post-transcriptional level.

The *NAC* family is one of the largest transcription factor families in plants and plays a pivotal role in the senescence program in many plant species, including *A. thaliana*, wheat, and rice [37]. For instance, *ANAC016*, *ANAC002*, and *ANAC032* facilitate senescence, whereas *ANAC083*, *ANAC041*, and *JUB1* inhibit senescence in *A. thaliana* [27,38,39,40]. In rice, *OsNAP*, *OsNAC2*, and *ONAC011* have been implicated in the modulation of aging- and dark-induced leaf senescence [41,42,43]. In the study of poplar, through RNA sequencing and gene co-expression network analysis, it was found that the alternative splicing variant of PtRD26, PtRD26IR, can delay leaf senescence. In the study of Arabidopsis thaliana, by using molecular biological and metabolomic methods, it was confirmed that RD26 is a key regulator of metabolic reprogramming during dark-induced senescence [44,45]. In the present study, through comprehensive bioinformatic screening, we found that *BrNAC104*, *BrNAC052*, and *BrNAC047* demonstrate remarkable upregulation under dark-induced senescence (Figure 3D, Figure S4). We also demonstrated that *BrNAC047*, *BrNAC052*, and *BrNAC104* actively modulate the senescence of NHCC leaves by influencing multiple metabolic processes (Figure 4 and Figure 6). Given that these candidate genes could synergistically induce the promoter activity of various SAGs, we examined their direct protein interactions using a yeast hybrid assay. However, no direct protein interactions were detected among BrNAC047, BrNAC052, and BrNAC104 (Figure S3). The results suggested that BrNAC047, BrNAC052, and BrNAC104 synergistically regulate leaf senescence in a parallel manner. In future agriculture production, various technical measures, such as gene-editing with CRISPR/Cas9 system, can be developed to delay leaf senescence by targeting these three genes. This contributes to cultivating new NHCC varieties with a delayed leaf senescence trait. On the other hand, inhibitors targeting these genes can also be developed to precisely suppress the expression of *BrNAC047*, *BrNAC052*, and *BrNAC104*, thus inhibiting the progression of leaf senescence in NHCC.

Light plays a crucial role in regulating plant growth and development by interacting with various phytohormones. Light influences the movement and distribution of auxin as well as the synthesis and signal transduction processes of cytokinin. Additionally, it modulates the inhibition of gibberellin biosynthesis and maintains the synthesis and efficacy of ethylene [46,47]. Among these intricate phytohormonal interaction networks, the association between ABA and light is unique and is crucial in the context of leaf senescence [48]. ABA has been shown to modulate the expression of SAGs via multiple signaling cascades [49,50,51]. In the present study, during the promoter element analysis of *BrNAC047*, *BrNAC052*, and *BrNAC104*, several phytohormone-responsive elements such as ABA-, auxin-, and JA-responsive elements were detected (Figure 4C). GO analysis of dark-induced DEGs further demonstrated that the dark-induced genes were conspicuously enriched in the ABA response pathway (Figure 2C). This result was consistent with our finding that *BrNAC047*, *BrNAC052*, and *BrNAC104* are induced by the ABA. Taken together, these results demonstrate that dark-induced leaf senescence in NHCC is mediated by ABA signaling.

## 4. Materials and Methods

### 4.1. Plant Materials and Growth Conditions

The NHCC variety used in the present study was previously developed by our laboratory and was stored at the Horticultural Research Institute of the Shanghai Academy of Agricultural Sciences. The plant materials were cultivated in incubators under a 16:8 h light/dark photoperiod, with a light intensity of approximately 60 µmol m^−2^ s^−1^ and a humidity level of 60%.

### 4.2. Dark Treatment

NHCC that had reached the five-true-leaf stage was subjected to treatment. Detached leaves were stored in the dark for 3 days, with light treatment serving as the control. The experiment was repeated three times.

### 4.3. Total RNA Extraction, Library Construction, and Sequencing

RNA extraction, library construction, and subsequent transcriptome sequencing were outsourced to Shanghai Majorbio Bio-Pharm Technology Co., Ltd. (Shanghai, China). The chinensis (Cultivar NHCC001) v1.0 Genome (Download Detail (njau.edu.cn)) was adopted as the reference genome. DESeq2 was employed for differential expression analysis. Genes showing a log2 (fold change) value ≥ 1 and adjusted *p*-value < 0.05 were designated as differentially expressed genes (DEGs).

### 4.4. Measurements of Chlorophyll Content and the F_V_/F_m_ Ratio

Positionally matched leaves from the control and experimental groups were examined to measure the maximum photochemical efficiency (F_V_/F_m_) of Photosystem II (PSII). Briefly, the leaves were placed in darkness for approximately half an hour, and subsequently, F_V_/F_m_ was determined using a calibrated chlorophyll fluorescence imaging system (WALZ IMAG—MAX/L, Effeltrich, Germany). The chlorophyll content was examined with a chlorophyll meter.

### 4.5. DAB and NBT Staining

NHCC leaves were stained for detecting reactive oxygen species (ROS) accumulation, as described previously [52]. The leaves were incubated in a 3,3′-diaminobenzidine (DAB) (1 mg/mL [pH 3.8]) and NBT (nitro blue tetrazolium) staining solution (0.5 mg/mL [pH 7.8]) for 6 h away from light. Subsequently, the leaves were immersed in 95% ethanol to remove chlorophyll, preserved in 95% ethanol, and photographed using a camera.

### 4.6. Virus-Induced Gene Silencing (VIGS)

First, 40-bp sequences from the coding region of *BrNAC047*, *BrNAC052*, and *BrNAC104* were selected and reverse-complemented to obtain 80-bp palindrome sequences. The sequences used for gene silencing are listed in Supplementary Table S1. The *BrNAC047*-silencing vector (PTY-*BrNAC047*), *BrNAC052*-silencing vector (PTY-*BrNAC052*), and *BrNAC104*-silencing vector (PTY-*BrNAC104*) were synthesized and constructed using Gene-Script. Two-week-old NHCC seedlings were used for the VIGS assay. PTY-*BrNAC047*, PTY-*BrNAC052*, PTY-*BrNAC104*, and PTY (control vector) (Kingsray Biotechnology Co., Ltd., Nanjing, China) were coated with gold powder and inserted into NHCC leaves using a GJ-1000 gene-gun (Zhejiang Xinzhi, Taizhou, China), as described previously [53]. One month later, qPCR was conducted to examine the gene silencing efficacy of these constructs in NHCC leaves.

### 4.7. Determination of Antioxidant Activity and Soluble Sugar Content

The soluble sugar and proline content of NHCC, as well as the malondialdehyde (MDA), peroxidase (POD), catalase (CAT), and superoxide dismutase (SOD) activities, were determined using commercial kits (Comin, Item No. POD-1-Y, CAT-1-W, SOD-1-W, BCAP-1-W, Suzhou, China) according to the manufacturer’s instructions.

### 4.8. Dual-Luciferase Assay

The promoter sequences of SAGs were cloned into the pGreenII 0800-LUC vector. Meanwhile, the coding sequences of the upstream transcription factors were inserted into the PCHF3 vector [54]. Tobacco leaves were infected with a transformed *Agrobacterium* strain (GV3101: pSoup) using a 9:1 *v*/*v* (effector:reporter) mixture. Empty PCHF3 plasmids were utilized as the negative control. Following 2 days of incubation, the Dual-Luciferase Reporter Assay System (TransGen, Beijing, China) was used to examine Firefly and Renilla luciferase activities. Fluorescence was monitored using a Synergy two multi-mode microplate reader (Bio-Tek, Winooski, VT, USA) according to the manufacturer’s instructions.

### 4.9. Yeast Two-Hybrid Assay

Full-length genes were amplified with TaKaRa Primer STAR Max DNA polymerase. *BrNAC047* and *BrNAC052* were added into a pGBKT7 vector, whereas *BrNAC104* was added into a pGADT7 vector. The plasmids were co-transformed into the yeast strain AH109, and single colonies were resuspended and grown on SD/−Leu/−Trp and SD/−Leu/−Trp/−Ade/−His medium for further analysis.

### 4.10. qRT-PCR Analysis

RNA was reverse transcribed into cDNA using the PrimeScript RT Master Mix Kit (TaKaRa, Kyoto, Japan). A 20 µL qPCR reaction mixture containing 1 µL of cDNA (250 ng/mL), 1 µL each of upstream and downstream primers (10 μm), and 10 µL of TransStart Top Green qPCR SuperMix was prepared, and the final volume was brought up to 20 µL using ddH_2_O. Primers were synthesized by Qingke Biotechnology Co., Ltd. (Beijing, China) and are listed in Table S1.

## 5. Conclusions

In this study, BrNAC047, BrNAC052, and BrNAC104 were identified as key promoters of dark-induced senescence in NHCC. These transcription factors were found to directly target the promoters of certain SAGs. With regard to upstream signals, our findings suggested that ABA may be involved in the dark-induced leaf senescence of NHCC by regulating the expression of *BrNAC047*, *BrNAC052*, and *BrNAC104*. Hence, this study highlights the role of these three key regulators and provides valuable molecular targets for delaying leaf senescence in NHCC. In the future, more *BrNAC047*, *BrNAC052*, and *BrNAC104* constructs should be generated to obtain genetic data for elucidating the mechanism of dark-induced leaf senescence in NHCC.

## Data Availability

The datasets presented in this study can be found in online repositories. The names of the repository/repositories and accession number(s) can be found below: http://www.ncbi.nlm.nih.gov/bioproject/1181662, PRJNA1181662, accessed on 4 November 2024.

## Supplementary Materials

The supporting information can be downloaded at: https://www.mdpi.com/article/10.3390/ijms26052340/s1.
