# Supplementary material for: NAC047/052/104 Synergistically Regulate the Dark-Induced Leaf Senescence in Non-Heading Chinese Cabbage
Source: Int J Mol Sci. 2025 Mar 6;26(5):2340. doi: 10.3390/ijms26052340 (PMC11900949; doi:10.3390/ijms26052340)
Supplement: Supplementary file 1 [file ijms-26-02340-s001.zip › ijms-3436215-supplementary.pdf]

NAC047/052/104 synergistically regulate the dark-induced leaf senescence in non-heading Chinese cabbage

Bing Yang[1,2,†], Dingyu Zhang[2,†], Zitong Meng[1,2], Yijiang Yin[2], Xiao Yang[2], Mengqin Cao[1,2], Ruixin Li[2], Yishan Song[1,*], and Hongfang Zhu[2,*]

[1]College of Food Science and Technology, Shanghai Ocean University, Shanghai 201306, China

[2]Shanghai Key Laboratory of Protected Horticultural Technology, Horticultural Research Institute, Shanghai Academy of Agricultural Sciences, Shanghai, 201403, China

[*]Corresponding author: yssong@shou.edu.cn (Yishan Song); zhuhongfang@saas.sh.cn (Hongfang Zhu);

[†] These authors contributed equally to this work.

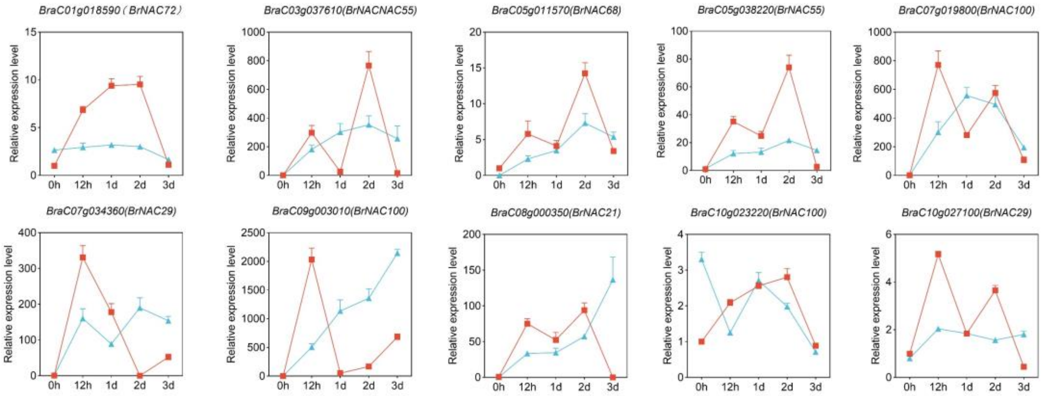

Figure S1 Transcriptional levels of NAC family genes

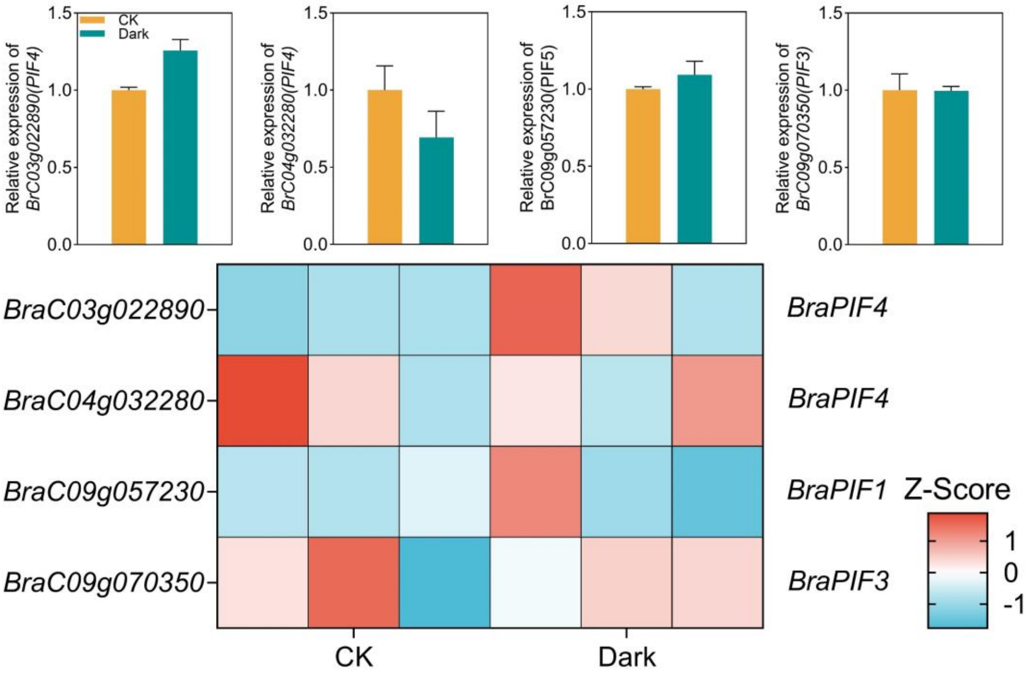

Figure S2 Relative expression levels of PIF3/4/5 genes in the dark

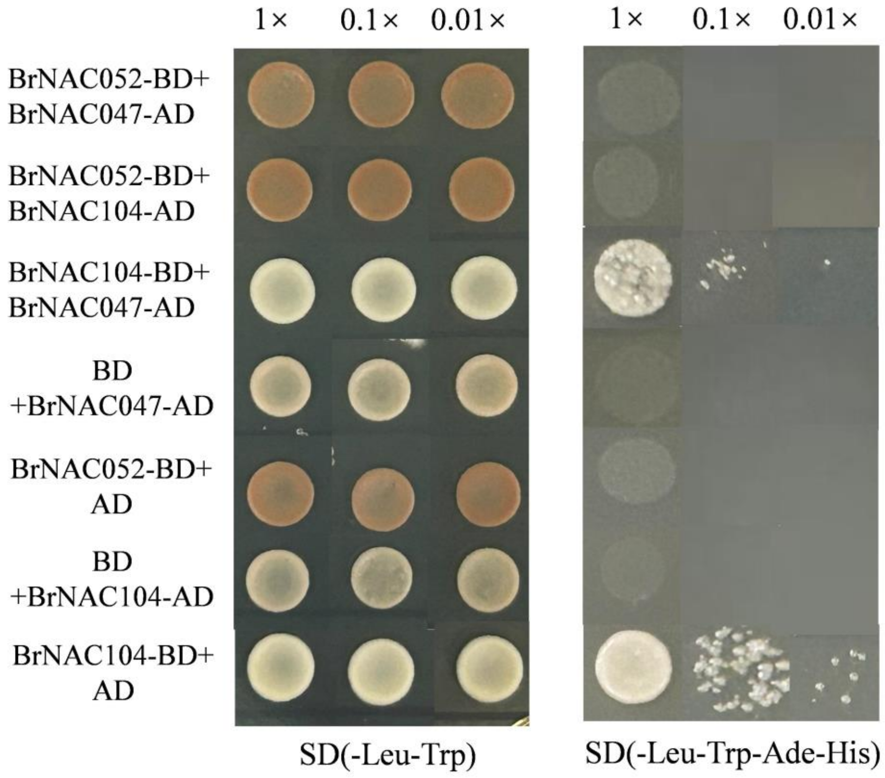

Figure S3 Proteins interacting with BrNAC047, BrNAC052, and BrNAC104 in yeast

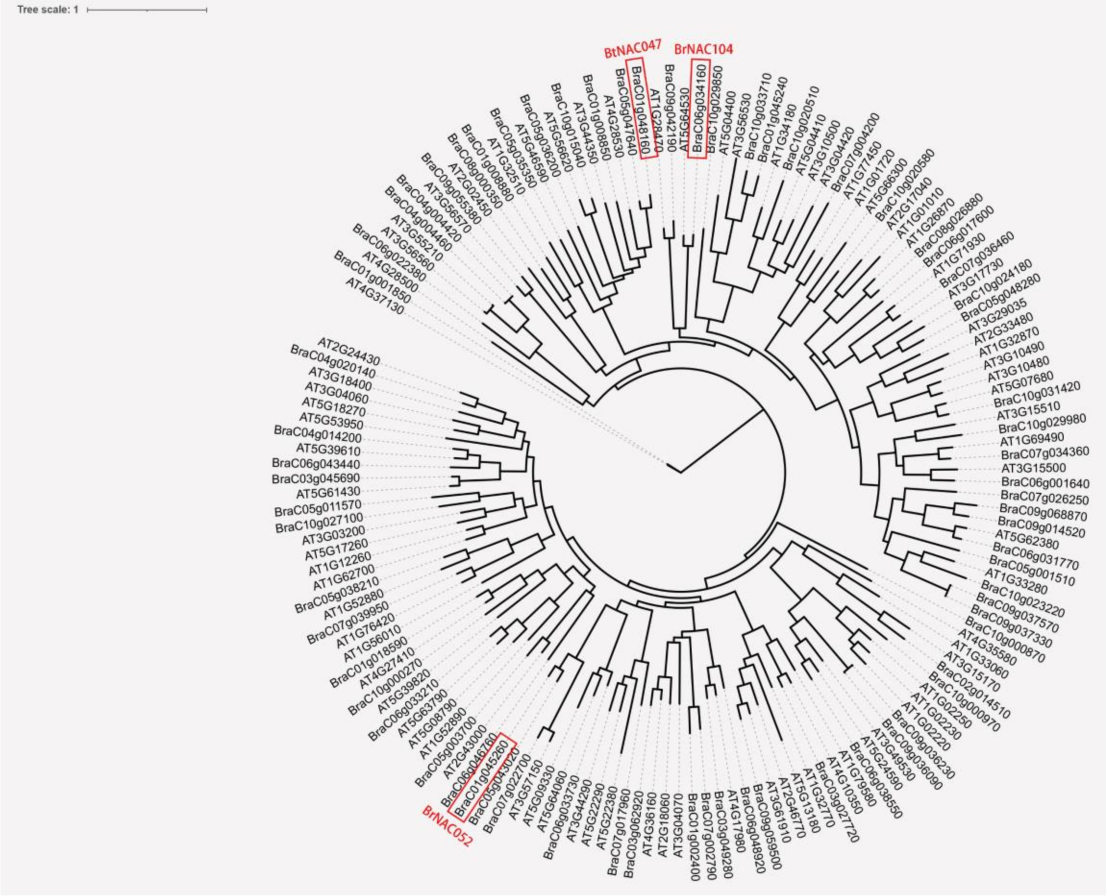

Figure S4 Evolutionary tree analysis of all NAC genes in NHCC.

Table S1. Primer sequences used in the present study.

Gene ID	Gene name	Forward sequence	Reverse sequence
BraC01g048160	*NAC047*	TGAGGCTGGATGATTGGGTT	CTGCTGGCGTTTAAGGGTTT
BraC01g045260	*NAC052*	AACGTGAGGAGGCAGATGAA	TTGTTGTTGTGGACGAGCAG
BraC06g034160	*NAC104*	ACGAGCCAATCTTCACAAGC	TAGAACCACGGCCTGATCTC
BraC01g018590	*NAC72*	GAAAGCTCCAAAAGGCACGA	GACATGTTCGTGCTCTGGTC
BraC03g037610	*NAC55*	GCAATCAAGCGCACAAAAGG	CGTAACGGACACTTGGAACG
BraC05g011570	*NAC68*	CTGTAAAGCCGAACCATGGG	CCCAATTGGTTCTTGTCCCG
BraC05g038220	*NAC55*	AAGTTTGGGTTATGCTGCCG	CGTGTAAATAGCCACCACCG

BraC07g019800	*NAC100*	TCCGACCAAGAAACCGAAGA	TAAACCGGTGGAGTCAGGAC
BraC07g034360	*NAC29*	GCCCTGTCTCGATCATACCA	AATAACCGGAAACAGCTGCC
BraC08g000350	*NAC21*	AGAAAGGGCAAGCTTGTTGG	GGAAGAGAAGCGGAGACTGT
BraC09g003010	*NAC100*	CCAATTGGGTCATGCACGAA	TTGATCCCTGAACCGAGCTT
BraC10g023220	*NAC100*	ATCCTGCTACGACAACCCAA	AAGTCGTGTTGTTCGGGTTG
BraC10g027100	*NAC29*	GCACAAGGGAAGCCAAGTAC	CGTGCATGATCCAATCGGTT
